# The Emerging Role of N-Acetylaspartate in Cancer

**DOI:** 10.3390/ijms27146105

**Published:** 2026-07-08

**Authors:** Yongzi Wu, Wenjuan Luo, Linbo Yao, Wei Huang, Shiyu Liu

**Affiliations:** 1 West China Centre of Excellence for Pancreatitis, Institute of Integrated Traditional Chinese and Western Medicine, West China-Liverpool Biomedical Research Centre, West China Hospital, Sichuan University, Chengdu 610041, China; yongziwu@163.com (Y.W.); wenjuanluo@wchscu.cn (W.L.); linbo_yao108@163.com (L.Y.); 2 West China Biobank, West China Hospital, Sichuan University, Chengdu 610041, China

**Keywords:** N-acetylaspartate, N-acetyltransferase 8-like, tumor metabolism, immune evasion

## Abstract

N-Acetylaspartate (NAA), historically considered a brain-restricted neuro-metabolite, has emerged as a pivotal regulator in cancer biology. Governed by the biosynthetic enzyme N-acetyltransferase 8-like (NAT8L), the NAA axis exerts notable biological effects in malignancies. This review delineates the NAA axis as a context-dependent metabolic rheostat that is strategically hijacked by malignancies to orchestrate growth and immune evasion. Rather than a passive bystander, the NAA axis functions through distinct, histology-specific paradigms: it either serves as a metabolic reservoir to fuel lipid biomass expansion or is suppressed to redirect aspartate flux toward nucleotide biosynthesis, depending on the tumor’s unique genetic and metabolic bottlenecks. Beyond cell-intrinsic reprogramming, tumor-derived NAA is increasingly linked to immune evasion by impairing cytotoxic lymphocyte function and driving pro-tumorigenic M2-like macrophage polarization. By reconciling these disparate oncogenic roles, this review highlights the NAA axis as an important node of metabolic plasticity and evaluates its potential utility as a circulating biomarker and a stratifiable therapeutic target in diverse human malignancies.

## 1. Introduction

A significant paradigm shift in cellular biology has redefined intermediate metabolites from passive energy substrates into potent signaling molecules that are associated with cell fate and epigenetic landscapes [1,2,3,4]. Implicit in this shift is the understanding that by coupling metabolic reprogramming with chromatin modifications, these metabolites drive diverse disease progressions, ranging from malignancy to systemic inflammation [5,6,7,8,9]. N-acetylaspartate (NAA) exemplifies this functional evolution [10,11]. Traditionally, NAA has been recognized as a specific neuronal marker and one of the most abundant amino acid derivatives in the mammalian central nervous system (CNS) [12,13,14,15]. NAA is synthesized within neuronal mitochondria by N-acetyltransferase 8-like (NAT8L) and subsequently shuttled to glial cells for hydrolysis by aspartoacylase (ASPA) [16,17,18]. Historically, this strict spatial compartmentalization restricted NAA research to the neurosciences, where it was regarded primarily as a neuronal osmolyte or a local carbon donor for myelin lipid synthesis [19,20,21]. The clinical significance of the NAA axis is best illustrated by Canavan disease, a fatal leukodystrophy where ASPA mutations lead to pathological NAA accumulation and severe neurotoxicity [22,23,24,25,26]. Conversely, early declines in cerebral NAA levels have long served as a diagnostic marker for neuronal loss in neurodegenerative disorders [27,28,29,30].

However, emerging evidence has challenged this traditional view by revealing that the NAA axis is pervasive in peripheral tissues. Furthermore, this axis is frequently dysregulated to promote malignant progression [31,32]. Yet, as empirical data proliferate, a critical interpretive gap has emerged: the seemingly contradictory observations of both elevated and diminished NAA levels across diverse malignancies. This raises a fundamental challenge for the field: Is the NAA axis a de facto oncogenic engine, a tumor-suppressive barrier, or a mere metabolic bystander? To resolve this paradox, we propose that the NAA axis functions as a context-dependent metabolic rheostat. Its pathological output is not stochastic; rather, it is precisely dictated by the tissue-specific metabolic bottlenecks and the distinct genetic aberrations, such as Ras homolog family member C (RhoC) amplification, characterizing each tumor lineage.

Accordingly, this review seeks to consolidate current understanding of the peripheral NAA axis into a cohesive conceptual framework within the context of oncology. We first provide an overview of the molecular pathways and regulatory factors that potentially drive the divergent expression of the NAA axis in different malignancies. Building on these observations, we explore the emerging pathophysiological logic behind high-NAA versus low-NAA states, discussing how these distinct metabolic profiles may offer adaptive advantages to the tumor. Furthermore, we examine the preliminary evidence regarding circulating NAA, considering its potential, as well as current limitations, as a non-invasive indicator of tumor burden. Finally, we discuss theoretical therapeutic opportunities tailored to varying NAA landscapes, ranging from the modulation of NAT8L activity to the strategic targeting of the immune microenvironment. By synthesizing these diverse perspectives, we propose that the NAA axis represents a noteworthy regulatory node of metabolic plasticity, potentially offering new avenues for understanding and addressing therapeutic resistance in cancer.

## 2. The Canonical NAA Axis: Baseline Physiology in the CNS

### 2.1. Molecular and Biochemical Foundations of the NAA Axis

The biological efficacy of the NAA axis is dictated by the precise enzymatic machinery of NAT8L and ASPA. NAT8L, a member of the general control nonderepressible 5-related N-acetyltransferase superfamily, catalyzes NAA synthesis [33,34]. Structurally, NAT8L is a membrane-bound protein characterized by a unique hydrophobic loop (residues 119–148) that anchors it to mitochondrial or endoplasmic reticulum membranes [33,35]. Its catalytic activity relies on conserved residues such as Arg^81^ and Glu^101^, which facilitate the orientation of aspartate and the subsequent acetyl transfer. Kinetic studies indicate that NAT8L catalytic efficiency is fundamentally limited by the availability of its precursors, acetyl coenzyme A (acetyl-CoA) and aspartate [35,36]. Recently, an additional layer of post-transcriptional regulation has been identified. Under conditions of oxidative stress, as occurs in multiple sclerosis pathology, reactive oxygen species induce selective guanosine oxidation within NAT8L mRNA to form 8-oxoguanosine [37]. This oxidative modification impairs its translation efficiency, leading to a marked reduction in NAT8L protein levels and consequent NAA deficiency [37]. This mechanism directly couples cellular redox status to NAA synthesis, independent of classical post-translational modifications.

In addition, the catabolic arm of the NAA axis is governed by ASPA, a specialized zinc-dependent metallopeptidase that functions as a 36 kDa homodimer in solution [38,39]. Each subunit consists of an N-terminal domain and a C-terminal domain, with a catalytic center containing a zinc ion coordinated by conserved histidine and glutamate residues, which is essential for polarizing the amide bond of NAA to facilitate its hydrolysis into acetate and aspartate [40,41]. However, ASPA exhibits limited substrate specificity. The Michaelis constant (*K*_m_) for NAA is approximately 0.36 mmol/L, but it shows even higher affinity (lower *K*_m_) for certain NAA analogs, such as N-dichloroacetyl-L-aspartate and N-trifluoroacetyl-L-aspartate [42]. Furthermore, ASPA activity is modulated by complex allosteric mechanisms. It exhibits self-activation at low NAA concentrations (<0.3 mmol/L) and self-inhibition at high concentrations (>1 mmol/L), suggesting the existence of additional regulatory binding sites that maintain NAA levels within a narrow physiological range [43]. Zn^2+^ is an indispensable structural and catalytic cofactor for ASPA. However, when present in excess, Zn^2+^ can catalyze the Haber-Weiss and Fenton reactions, generating reactive oxygen species that impair mitochondrial integrity. In SN56 cholinergic cells, Zn^2+^-induced oxidative stress leads to a depletion of the mitochondrial acetyl-CoA pool, which blunts NAT8L-mediated NAA production [36]. This mechanism effectively couples the NAA axis with cellular energy homeostasis and oxidative metabolism. Regarding other redox-active metals (Fe, Cu), no direct regulation of NAT8L or ASPA activity has been reported. Nevertheless, Fe^2+^ and Cu^2+^ are also capable of catalyzing Fenton/Haber-Weiss chemistry. In principle, they could indirectly affect NAA synthesis by promoting oxidative stress and depleting acetyl-CoA, a possibility that warrants future investigation.

### 2.2. Baseline Physiology of the NAA Axis

**Figure 1:**
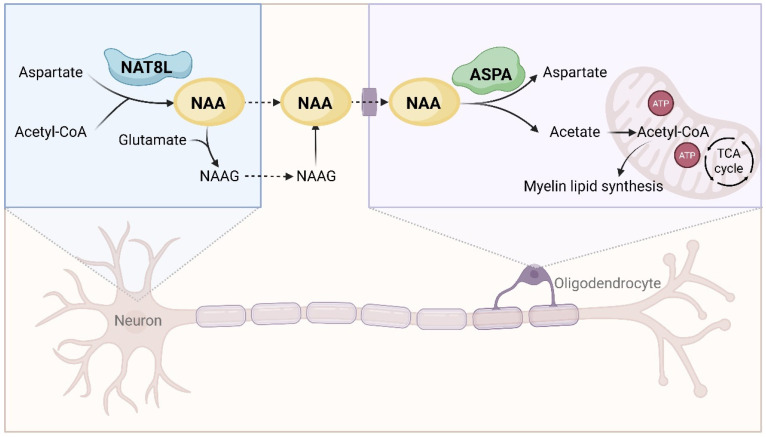
The canonical NAA metabolic shuttle between neurons and oligodendrocytes in the CNS. NAA operates as a fundamental driver of neuro-glial communication through a spatially compartmentalized metabolic axis. Within the neuron (**left** panel), NAA is synthesized from aspartate and acetyl-CoA by the enzyme NAT8L. A portion of this NAA combines with glutamate to form NAAG. Because neurons lack the primary catabolic enzyme, NAA is exported into the extracellular space and shuttled to adjacent oligodendrocytes (**right** panel). Inside the oligodendrocyte, the enzyme ASPA hydrolyzes NAA into aspartate and acetate. This liberated acetate enters the mitochondria and is converted into acetyl-CoA, which fuels the TCA cycle for ATP production and provides essential metabolic substrates for myelin lipid biosynthesis, thereby supporting continuous neuro-glial crosstalk and neuronal integrity. Abbreviations: NAA, N-acetylaspartate; NAT8L, N-acetyltransferase 8-like; ASPA, Aspartoacylase; NAAG, N-acetylaspartylglutamate; Acetyl-CoA, Acetyl coenzyme A; TCA, Tricarboxylic acid; ATP, Adenosine triphosphate.

In CNS, NAA operates as a fundamental driver of neuro-glial communication (Figure 1). It is synthesized within neuronal mitochondria from acetyl-CoA and aspartate by the enzyme NAT8L [36,44,45], reaching high intracellular concentrations of up to 20 mM [46,47]. Crucially, NAA catabolism is geographically segregated from its synthesis [48]. The primary catabolic enzyme, ASPA, is conspicuously absent in neurons but is robustly expressed in oligodendrocytes and microglia [49,50]. This strict spatial compartmentalization necessitates a metabolic shuttle where neuronally synthesized NAA is exported to glial cells for hydrolysis [51,52,53]. This continuous metabolic crosstalk is vital for maintaining local energy homeostasis and overall neuronal integrity.

Beyond serving as a simple metabolic intermediate, the NAA axis exerts pleiotropic regulatory effects across the CNS. Traditionally, ASPA-mediated hydrolysis of NAA was thought to primarily supply acetate for myelin lipid biosynthesis [54,55,56]. However, recent evidence reveals that NAA also functions as a potent epigenetic signaling molecule. For instance, neuron-derived NAA modifies histone methylation and activates histone deacetylases (HDACs) to directly promote oligodendrocyte maturation and myelination [57,58]. Furthermore, physiological levels of NAA provide a continuous neuroprotective shield. It acts as a molecular stabilizer with potent anti-glycating properties, effectively preventing the pathological aggregation of neurodegenerative proteins such as amyloid-β and α-synuclein [59,60,61,62,63]. Concurrently, extracellular NAA modulates neuroinflammation by epigenetically repressing inflammatory cytokine transcription, thereby maintaining microglia in a surveillant, homeostatic state [64]. Together, these classical CNS functions establish the NAA axis as a potential epigenetic and metabolic regulator, providing a foundational baseline for understanding its diverse emerging roles in peripheral tissues.

## 3. The Pathological Role of the NAA Axis in Cancer

### 3.1. Metabolic Duality: The NAA Axis as a Context-Dependent Rheostat

**Figure 2:**
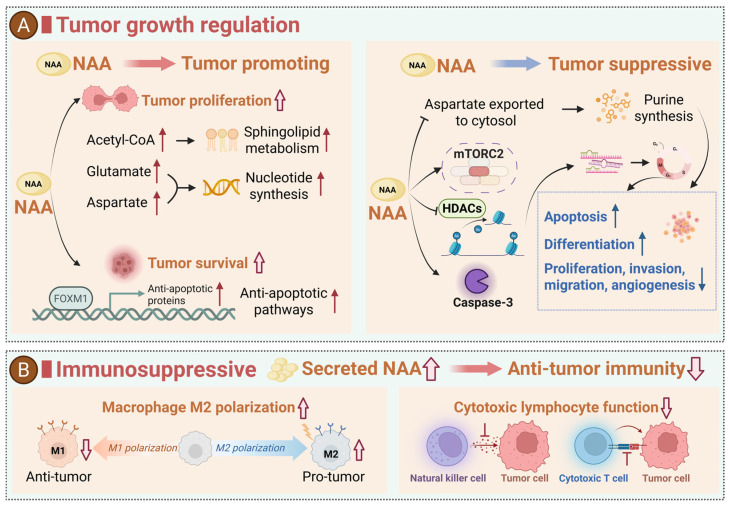
The supposed multifaceted roles of NAA in cancer. (**A**) The NAA metabolic axis exhibits highly context-dependent functions in oncology. In tumor-promoting contexts (**top left**), NAA fuels lipid and nucleotide synthesis and enhances survival via FOXM1 signaling. In tumor-suppressive contexts (**top right**), NAA depletion frees aspartate for purine synthesis, while exogenous NAA can suppress growth via mTORC2, HDAC inhibition, and apoptosis. (**B**) Tumor-secreted NAA promotes M2 macrophage polarization and impairs natural killer (NK) cells and cytotoxic T cells, driving immune evasion. Abbreviations: NAA, N-acetylaspartate; Acetyl-CoA, Acetyl coenzyme A; FOXM1, Forkhead box protein M1; mTORC2, Mammalian target of rapamycin complex 2; HDACs, Histone deacetylases; TME, Tumor microenvironment; NK, Natural killer.

The NAA axis functions as a pleiotropic regulatory node in cancer biology, transcending its historical perception as a mere metabolic byproduct (Figure 2A). Rather than exerting a uniform effect, the axis operates as a context-dependent rheostat that either fuels biomass generation or exerts suppressive signaling based on specific lineage wiring and metabolic bottlenecks. This metabolic duality manifests as two distinct clinical phenotypes, specifically the “High-NAA” engine and the “Low-NAA” rerouting strategy.

#### 3.1.1. The High-NAA Phenotype: A Metabolic Engine Fueling Tumor Aggression

Metabolic reprogramming is a fundamental hallmark of cancer, enabling malignant cells to meet the immense energetic and biosynthetic demands of rapid proliferation [65,66]. In several malignancies, the aberrant upregulation of NAT8L and accumulation of NAA confer distinct metabolic advantages that directly sustain tumor proliferation and survival.

Oncogenic signaling pathways actively hijack NAA biosynthesis to establish a metabolic engine that directly fuels tumor growth and survival. For instance, in inflammatory breast cancer (IBC), the oncogene RhoC acts as an upstream master regulator that fundamentally rewires glutamine metabolism to mass-produce NAA [67]. Importantly, rather than allowing NAA to accumulate as a passive byproduct, RhoC-driven tumors actively exploit this NAA pool as a dynamic metabolic reservoir to sustain malignant proliferation [67]. Under nutrient-scarce conditions typical of the tumor microenvironment, cancer cells mobilize and break down this stored NAA to release abundant acetyl groups [67]. These liberated building blocks directly fuel downstream lipid biomass expansion and maintain energetic homeostasis, thereby preventing cell death and actively driving aggressive tumor progression [67]. This profound dependency on NAA for survival is similarly validated in non-small cell lung cancer (NSCLC), where glutaminolysis specifically supplies the aspartate moiety required for NAA synthesis, and glutamine deprivation completely abolishes NAA production [68]. Consequently, upstream regulators strategically manipulate the NAA axis to secure a robust biosynthetic foundation that guarantees tumor growth and adaptability [67,68].

Once synthesized, NAA functions as a stable metabolic reservoir for acetyl groups. Recent multi-omics analyses have revealed a pronounced accumulation of NAA and N-acetylaspartylglutamate (NAAG) in castration-resistant prostate cancer (CRPC) tissues [69]. This accumulation is accompanied by the upregulation of N-acetylated alpha-linked acidic dipeptidases, which convert NAAG to NAA [69]. In CRPC, elevated NAA is hypothesized to provide a vital pool of acetyl-CoA to support upregulated sphingolipid metabolism. Additionally, it supplies the glutamate and aspartate necessary for nucleotide synthesis during active tumor growth [69]. Similarly, glioma stem-like cells actively hijack NAA and NAAG as a critical source of acetate for lipid synthesis to accelerate tumor growth [70].

The functional importance of this axis in tumor progression has been supported by genetic ablation studies. Specifically, silencing *NAT8L* in models of both ovarian cancer and melanoma profoundly reduces cancer cell viability and proliferation [32]. Underscoring the direct dependence of these tumors on this pathway, these inhibitory effects can be rescued by the exogenous supplementation of NAA [32]. Mechanistically, the depletion of *NAT8L* downregulates anti-apoptotic pathways mediated through forkhead box protein M1, thereby restricting tumor growth in vivo [32]. Beyond these survival signaling networks, stable isotope labeling experiments in A549 lung adenocarcinoma cells have indicated that *NAT8L* silencing directly inhibits cancer cell proliferation by disrupting essential metabolic flux pathways [71]. Collectively, these mechanistic insights and preclinical findings highlight the NAA axis as an emerging metabolic regulator that contributes to tumor survival, proliferation, and metabolic plasticity.

Elevated NAA levels and the corresponding upregulation of its biosynthetic enzyme NAT8L are observed in additional solid tumors. Rather than acting as isolated markers, they function together as an integrated oncogenic axis indicating aggressive disease and poor clinical prognosis.

At the metabolite level, tumor NAA concentrations reach micromolar levels in NSCLC while remaining low in healthy individuals, enabling blood-based detection that correlates directly with tumor burden [68]. This metabolic accumulation is closely tied to disease progression in other malignancies as well. For example, NAA levels in ovarian cancer ascites correlate closely with advanced disease stages [31]. The prognostic value of this axis is most strikingly illustrated in CRPC. In these patients, a distinct NAA-related gene signature is associated with a nearly twofold reduction in median overall survival (70 months versus 131 months), as well as higher metastasis rates and elevated Gleason grades [69]. Furthermore, in malignancies where direct clinical tracking of NAA levels is currently limited, the upstream drivers of its biosynthesis serve as crucial prognostic proxies. In IBC, the oncogene RhoC is notoriously overexpressed and strongly associated with aggressive metastatic phenotypes and diminished survival [67,72,73].

The prognostic significance of this metabolic phenotype is further reinforced at the transcriptomic and genetic levels through the evaluation of NAT8L. In high-grade serous ovarian cancer (HGSOC), high tumoral NAA concentrations coupled with elevated NAT8L mRNA expression levels are predictive of significantly worse overall survival [32]. Broadening this perspective, pan-cancer transcriptomic analysis further demonstrates that *NAT8L* is upregulated in 6 malignancies, including lung adenocarcinoma, lung squamous cell carcinoma, liver hepatocellular carcinoma, thyroid carcinoma, uterine corpus endometrial carcinoma, and cholangiocarcinoma [74]. Notably, its expression is significantly associated with a shorter progression-free interval, disease-specific survival, and overall survival in lung squamous cell carcinoma [74]. Beyond simple expression levels, *NAT8L* mutations are present in multiple cancer types, such as 1.5% in kidney chromophobe, 1.4% in colon adenocarcinoma, and 1.1% in colorectal adenocarcinoma, and its expression level effectively stratifies tumors with distinct mutation profiles [74]. For instance, *PBRM1*, *BAP1*, and *ARID1A* mutations are enriched in NAT8L-high cholangiocarcinoma, while *TTN*, *MVC16*, and *KMT2C* mutations are enriched in NAT8L-high kidney renal papillary cell carcinoma [74]. These co-occurring genetic aberrations highlight the context-specific nature of NAA axis dysregulation and its potential as a stratified prognostic indicator.

#### 3.1.2. Low-NAA Phenotype: Driving Proliferation via Metabolic Rerouting

Paradoxically, some cancers strategically downregulate NAT8L, resulting in a low NAA state. In these contexts, the axis acts as a functional tumor suppressor, and its dismantling confers a distinct metabolic advantage conducive to rapid proliferation.

The exploitation of the NAA axis is highly context-dependent. In hepatocellular carcinoma (HCC), tumors suppress NAT8L to bypass metabolic bottlenecks. NAT8L downregulation blunts mitochondrial NAA synthesis, causing an accumulation of unutilized aspartate that is subsequently exported into the cytosol via solute carrier family 25 member 13 (SLC25A13) [75]. This cytosolic aspartate redistribution then fuels the pentose phosphate pathway and purine biosynthesis to drive cell proliferation [75]. Experimental evidence confirms that NAT8L knockdown in HCC cell lines increases cytosolic aspartate and accelerates proliferation, proving that these tumors dismantle the NAA axis to liberate resources for rapid division. [75]. Thus, tumors dismantle the NAA axis to free up resources for rapid growth in HCC.

Complementing the observation that NAT8L downregulation promotes proliferation, exogenous NAA supplementation exerts potent anti-proliferative effects, particularly in neural-lineage malignancies. This suggests that in certain biological backgrounds, the loss of NAA is a prerequisite for rapid division. In the human catecholaminergic neuroblastoma cell line SH-SY5Y, exogenous NAA profoundly inhibits cell growth by inducing G1 phase cell-cycle arrest and promoting neuronal differentiation [76]. Similarly, in U87 malignant glioma cells, NAA treatment reduces HDAC mRNA levels and modulates the mammalian target of rapamycin complex 2 signaling complex to potentially suppress cancer progression [77]. These observations underscore that the low-NAA state is not merely a byproduct of metabolic rerouting, but may also serve to remove intrinsic inhibitory signals governed by the NAA axis.

The clinical implications of the NAA axis exhibit heterogeneity across various malignancies, reflecting its role as a metabolic rheostat. Pan-cancer transcriptomic analysis has revealed that *NAT8L* is significantly downregulated in 12 cancer types compared to normal tissues, including kidney renal clear cell carcinoma, breast invasive carcinoma, colon adenocarcinoma, and colorectal adenocarcinoma [74]. Notably, mechanistic understanding of this phenotype remains limited across diverse cancer types, warranting further investigation, particularly given transcriptomic data indicating NAT8L downregulation in multiple malignancies.

The clinical relevance of this axis is particularly evident in HCC, where the suppression of the NAA axis serves as a significant predictor of high-grade malignancy and poor patient outcomes. Specifically, low NAT8L expression and diminished NAA concentrations are strongly correlated with advanced histologic grades and significantly shorter overall survival [75]. Beyond its role as a prognostic marker, the NAA axis represents a stratifiable therapeutic vulnerability. Pan-cancer analysis suggests that axis downregulation correlates with altered immune infiltration patterns and tumor heterogeneity. These changes impact patient responsiveness to immunotherapy [74]. Furthermore, bioinformatic screening has identified that low NAT8L tumors exhibit hypersensitivity to specific small-molecule drugs, positioning the NAA axis as a promising predictive biomarker for personalizing metabolic intervention strategies [74]. Whether the link between axis downregulation and rapid division represents a universal clinical phenotype for other low-NAT8L cancers remains a compelling frontier for precision oncology. A comprehensive overview of these divergent expression patterns and their clinical implications across human malignancies is provided in Table 1.

In summary, the NAA axis operates as a context-dependent metabolic rheostat that dictates distinct tumor progression strategies based on lineage-specific bottlenecks. In the “High-NAA” phenotype, tumors accumulate a stable reservoir of NAA, as seen in NSCLC, IBC, HGSOC, and CRPC. Under nutrient-scarce or oncogene-driven conditions, cancer cells mobilize this stored pool to release acetyl groups that fuel downstream lipid biomass expansion and membrane synthesis. Conversely, tumors employ the opposite strategy in the “Low-NAA” phenotype. In HCC, cells strategically downregulate NAT8L to dismantle the NAA reservoir, preventing mitochondrial aspartate from being converted into NAA. The unutilized aspartate is then exported into the cytosol, where it enters the pentose phosphate pathway and purine biosynthesis to drive rapid cell division. Understanding these divergent metabolic dependencies clarifies the dualistic roles of the NAA axis and establishes a clear foundation for stratified, phenotype-specific therapeutic interventions.

### 3.2. Beyond Metabolism: NAA-Mediated Immunosuppression and TME Remodeling

While NAA has traditionally been recognized for its role in metabolic support, emerging evidence highlights its critical function as a potent signaling molecule. In the CNS, extracellular NAA epigenetically represses inflammatory cytokine transcription to maintain microglia in a homeostatic state, preventing excessive neuroinflammation [64]. Intriguingly, malignant cells appear to co-opt this central immunomodulatory logic: they actively secrete NAA to manipulate the tumor microenvironment (TME), effectively mimicking the anti-inflammatory mechanisms normally restricted to the CNS to establish a state of immune resistance [78]. For instance, in human epidermal growth factor receptor 2-positive breast cancer, tumors overexpress NAT8L and release NAA, which impairs anti-tumor immunity by inhibiting the cytotoxicity of natural killer cells and CD8^+^ T cells [79]. At the molecular and cellular level, NAA disrupts the formation of the immunological synapse by promoting E1A binding protein p300/CREB-binding protein-associated factor-induced acetylation of lamin A-lysine 542, which subsequently inhibits integration between lamin A and the inner nuclear membrane protein Sad1 and UNC84 domain-containing 2, ultimately impairing the polarization of lytic granules [79].

Beyond its detrimental effects on cytotoxic lymphocytes, NAA also acts as a regulator of innate immune cells, particularly concerning macrophage phenotypic plasticity. In highly invasive models of ovarian cancer, cancer cells that exhibit an addiction to extracellular glutamine release substantial amounts of NAA into the TME [31]. Molecular docking analysis has demonstrated that this tumor-derived NAA can bind to the agonist-binding domain of the N-methyl-D-aspartate (NMDA) receptor, acting as a competitive inhibitor [31]. Experimentally, treatment with a physiologically relevant dose of 10 μM NAA was shown to functionally inhibit NMDA receptor signaling, which acts synergistically with interleukin-10 to enforce glutamine synthetase expression, thereby driving macrophages toward a tumorigenic, M2-like phenotype [31]. These findings provide a structural and dose-dependent basis for NAA as a direct signaling mediator in the TME, although further biophysical studies are required to fully map the binding kinetics in vivo. Conversely, therapeutically targeting and reducing NAA levels holds significant promise for restoring anti-tumor immune function. As a proof of concept for pharmacological intervention, treatment with Shiyiwei Shenqi tablets in NSCLC effectively suppresses NAA accumulation by downregulating NAT8L and upregulating ASPA [80]. This metabolic modulation relieves nuclear factor kappa-B inhibition, successfully repolarizing macrophages toward an anti-tumor M1-like phenotype and subsequently reducing M2-driven angiogenesis and metastasis [80].

However, it should be noted that the evidence for NAA-mediated immunosuppression remains preliminary and warrants cautious interpretation. The mechanistic insights into NAA-mediated impairment of NK cells and CD8^+^ T cells, as well as macrophage polarization, are currently derived from a limited number of studies and lack independent validation across broader tumor types or experimental systems. Thus, while the NAA axis emerges as an intriguing immunomodulatory node, the current evidence should be considered as a foundation for hypothesis generation rather than a consolidated paradigm.

In addition, the impact of NAA on other critical immune populations, such as regulatory T cells, dendritic cells, or myeloid-derived suppressor cells, remains largely unexplored. Beyond these cellular populations, the capacity of tumor-derived NAA to remodel the TME also raises the question of whether NAA levels could modulate responses to contemporary immunotherapies. Elevated NAA could conceivably contribute to resistance against immune checkpoint inhibitors by fostering an immunosuppressive microenvironment. It might also theoretically limit chimeric antigen receptor T-cell therapy efficacy by compromising immunological synapse formation and lytic granule polarization. These possibilities, although speculative, warrant future investigation.

Collectively, tumor-derived NAA orchestrates robust immune evasion by simultaneously impairing cytotoxic lymphocyte function and promoting M2-like macrophage polarization (Figure 2B). Consequently, therapeutic blockade of the NAA metabolic axis offers a compelling strategy to reverse immunosuppression and remodel the TME.

### 3.3. Opportunities and Caveats of NAA as a Biomarker Candidate

The distinct biosynthetic profile of the NAA axis positions it as an intriguing candidate for diagnostics. Under physiological conditions, NAA is virtually undetectable in healthy lung epithelium, whereas NSCLC tumors synthesize it in massive quantities and actively secrete excess NAA into the systemic circulation, enabling blood-based detection [68]. However, to substantiate this proposal, the analytical and biological caveats must be critically appraised and the current evidence cautiously framed.

A primary analytical hurdle resides in the stark concentration discrepancy between compartments. Tissue NAA spans the micromolar range, whereas circulating concentrations are vastly lower, typically residing in the low nanomolar range [68]. This hundred-to-thousand-fold gradient introduces the risk of cross-study data incompatibility across platforms with divergent sensitivity. Liquid chromatography-tandem mass spectrometry (LC-MS/MS), operated in multiple reaction monitoring mode, enables direct analysis of highly polar metabolites with superior sensitivity [81,82]. Recent technological advances have further pushed detection limits. For instance, Zhou et al. achieved a limit of detection as low as 0.019–0.052 nM for NAA [83]. This was accomplished using in-tube solid-phase microextraction coupled with ultra-high-performance LC-MS/MS [83]. Single-center assays incorporating derivatization and stable isotope-labeled internal standards (e.g., ^13^C_4_-NAA) have also achieved excellent intra- and inter-day precision (relative standard deviations < 3%) [84]. In contrast, gas chromatography-mass spectrometry (GC-MS) offers robust chemical identification via commercial spectral libraries. However, its requirement for tedious chemical derivatization introduces analytical variance. Consequently, GC-MS may lack the sensitivity required for trace-level analysis of circulating compounds [85,86]. Although useful for broader metabolic mapping, untargeted metabolomics provides only relative quantification. Furthermore, it suffers from low metabolite annotation rates (10–20%) and exhibits poor inter-laboratory reproducibility due to matrix interferences [87,88]. Furthermore, although pre-analytical stability dictates data fidelity, recent methodological advancements incorporating sample derivatization and stable isotope internal standards have enhanced assay accuracy and compensated for pre-analytical fluctuations [84]. Nonetheless, systematic multi-center comparisons of inter-laboratory NAA quantification accuracy are currently lacking. These methodological discrepancies suggest that the reported 46% serum diagnostic sensitivity for NSCLC may reflect technical limitations of earlier assays as much as true biological variance [68].

Beyond analytical challenges, it must be emphasized that circulating NAA is unlikely to function as a stand-alone screening biomarker. Its modest sensitivity and lack of absolute cancer-specificity constrain stand-alone performance. Crucially, interpretation of circulating metabolites is heavily confounded by non-oncological disorders that disrupt systemic homeostasis. For instance, Parkinson’s disease is now increasingly recognized as a multisystem immune-metabolic disorder involving peripheral inflammation, altered metabolic pathways, exosome-mediated communication, and α-synuclein-related immune activation [89]. The systemic and multi-organ nature of such neurodegenerative and metabolic conditions can reshape peripheral metabolic profiles, introducing confounding variables for diagnostic specificity. Similarly, altered serum NAA levels have been detected under specific neurological conditions, such as ischemic stroke [90] and Alzheimer’s disease [91]. Moreover, NAA released from adipose tissue under specific metabolic conditions may confound interpretation in patients with metabolic comorbidities [92,93].

Besides these overlapping peripheral metabolic influences, fundamental validation criteria, including independent prognostic value, external multi-center validation, and demonstrated clinical utility, remain largely unexplored. At present, the available evidence supports NAA as a potential biomarker candidate rather than a clinically validated tool, and stronger claims should be avoided until larger validation studies are available. Thus, to optimize clinical utility and counteract these confounding factors, NAA should be deployed as part of rigorously designed metabolomic panels rather than relying on it as an isolated, standalone cancer marker. Alternatively, its primary clinical niche may lie in the longitudinal monitoring of tumor burden, therapeutic response, and early relapse detection, scenarios where its longitudinal correlation with clinical staging has already shown promise in NSCLC and ovarian cancer [31,68]. Future research must prioritize large-scale prospective trials under standardized pre-analytical and analytical workflows to systematically validate its diagnostic performance before clinical translation.

### 3.4. Stratified Therapeutic Strategies for Divergent NAA Phenotypes

The dualistic behavior of the NAA axis, characterized by its upregulation in certain malignancies (e.g., NSCLC, HGSOC) and its suppression in others (e.g., HCC), precludes a “one-size-fits-all” therapeutic approach. Instead, effective intervention must be guided by the tumor’s specific NAA status, employing a stratified strategy that aligns with the metabolic logic of each phenotype.

In high-NAA tumors where the NAA axis is hijacked as an oncogenic driver, the primary goal is to disrupt the supply of NAA-derived acetyl groups and reverse immune evasion. Preclinical evidence has demonstrated that *NAT8L* silencing significantly reduces cell viability in ovarian cancer, melanoma, and lung adenocarcinoma, effects that are specifically rescued by exogenous NAA [32,71]. A detailed summary of targeted interventions against the NAA axis and their key experimental findings across diverse preclinical models is provided in Table 2. Encouragingly, recent advances in small-molecule discovery have identified low-micromolar, non-carboxylic acid inhibitors of NAT8L that act competitively with respect to aspartate [34]. While these findings are promising, further rigorous preclinical validation is required. Alternative strategies for these tumors include targeting downstream pathways dependent on NAA-derived acetate, such as sphingolipid synthesis. Another approach involves employing NMDA receptor antagonists to block tumor-secreted NAA signaling, which subsequently repolarizes M2-like macrophages within the TME [31,69].

In low-NAA tumors, where NAT8L acts as a functional tumor suppressor, its downregulation liberates mitochondrial aspartate for cytosolic purine biosynthesis. While direct pharmacological restoration of NAT8L remains a significant challenge, a more feasible approach involves targeting the compensatory metabolic nodes created by this rerouting. For instance, blocking the frequently upregulated aspartate exporter SLC25A13 has been shown to impair proliferation in NAT8L-low preclinical HCC models [75]. Furthermore, repurposing established inhibitors of de novo purine synthesis may offer selective efficacy in NAT8L-low tumors that are addicted to aspartate-driven nucleotide expansion, a hypothesis that warrants clinical investigation.

Therefore, the pathophysiological advantage of high NAA is to support lipid synthesis and immune evasion, while the advantage of low NAA is to enable nucleotide synthesis via aspartate rerouting. By matching intervention strategies to the tumor’s specific NAA status, we can exploit the metabolic dependencies unique to each malignancy. Ultimately, this stratified approach resolves the longstanding confusion surrounding NAA’s dual role, allowing us to view the NAA axis not as a static marker, but as a context-dependent metabolic rheostat.

## 4. Insights from Systemic Metabolism: NAA Axis in Adipose Tissue

To fully realize the clinical potential of the NAA axis discussed above, it is imperative to contextualize these oncogenic findings within the broader landscape of systemic metabolism. The NAA metabolic axis is not exclusively restricted to the CNS or malignant tissues. It is also functionally operational in adipose tissue, where it plays a fundamentally analogous role by sequestering acetyl-CoA and aspartate into NAA to create a mobilized reservoir of acetyl groups for biosynthetic and energetic demands [94,95,96].

In brown adipose tissue, NAT8L synthesizes NAA from glucose-derived acetyl-CoA, effectively diverting carbon flux away from immediate oxidation [94]. This stored NAA can later be hydrolyzed by ASPA to release acetate, which is converted back to acetyl-CoA to fuel lipogenesis or the tricarboxylic acid cycle [94,95,97]. Thus, the enzymatic logic observed in high-NAA tumors, where NAT8L is upregulated to capture acetyl-CoA for biomass expansion, parallels the physiological buffering mechanism in normal adipose tissue, albeit under nutrient-responsive control rather than oncogenic dysregulation. A critical distinction lies in the fate of the synthesized NAA. In tumors, excess NAA is actively secreted to manipulate the immune microenvironment. In contrast, in adipose tissue, NAA primarily acts as an intracellular buffer. However, under specific ASPA-regulated conditions, it can enter the systemic circulation to modulate postprandial body temperature via pyrimidine synthesis pathways [92].

This metabolic parallel has two important implications that directly impact oncological research. First, it validates NAA as a conserved metabolic storage strategy across diverse cell types, rather than a mere cancer-specific aberration. Second, it raises a cautionary note for liquid biopsy development. Since circulating NAA reflects both tumor burden and the metabolic state of adipose tissue, which fluctuates with obesity, insulin resistance, and nutritional status, interpreting serum NAA as a cancer-specific marker requires accounting for these systemic confounders. This limitation further reinforces the necessity of integrating NAA into multi-metabolite panels.

Ultimately, the adipose NAA axis reinforces the concept of NAA as both a dynamic acetyl-CoA reservoir and a metabolite with potential endocrine properties. This systemic perspective does not diminish the rationale for targeting NAT8L in high-NAA cancers. Instead, it underscores the requirement for tissue-specific or stratified interventions to avoid disrupting metabolic homeostasis in healthy peripheral tissues, thereby setting the stage for the next phase of precision oncology.

## 5. Concluding Remarks and Future Perspectives

The scientific understanding of NAA has undergone a profound paradigm shift, evolving from its historical perception as a passive neuronal osmolyte strictly confined to the central nervous system into a sophisticated, systemic signaling molecule. By integrating recent advancements across oncology and immunometabolism, it is now evident that the NAA axis represents a critical bridge between mitochondrial metabolism and cellular identity in peripheral tissues. The strategic hijacking of this axis by malignant cells for nutrient scavenging and immune evasion highlights its pervasive influence on human health and disease, moving the discussion of this metabolite from the periphery to the center of cancer research.

Despite these advancements, several fundamental questions provide fertile ground for future investigation. A primary challenge lies in deciphering the deep-seated mechanisms driving the metabolic dichotomy of the NAA axis across different cancer types. While it is established that the axis promotes progression in many solid tumors through lipogenic support while exerting suppressive effects in specific hepatic and neuroendocrine contexts, the precise genetic and epigenetic drivers behind these divergent high-NAA and low-NAA phenotypes remain largely opaque. Determining whether these variations are a result of lineage-specific metabolic bottlenecks or distinct oncogenic signaling rewiring will be essential for the rational design of context-appropriate therapeutic strategies targeting this axis that do not inadvertently disrupt systemic homeostasis. Furthermore, while the NMDA receptor has been identified as a target for NAA in tumor-associated macrophages, the specific membrane transporters and downstream signaling receptors across other peripheral organs remain largely undefined, necessitating a more comprehensive mapping of the systemic NAA interactome.

In terms of clinical translation, it must be acknowledged that empirical evidence establishing NAA as a robust prognostic or diagnostic biomarker remains limited. While this review provides an initial framework for the clinical potential of the NAA axis, current data are often derived from small-scale cohorts or preclinical models. Serum NAA, despite showing promise as a non-invasive surrogate for monitoring treatment response, faces significant hurdles due to its modest sensitivity and the confounding metabolic contributions from adipose tissue. Therefore, the clinical utility of NAA cannot yet support its use as a standalone diagnostic tool. Future research must prioritize large-scale prospective clinical studies to validate these preliminary findings and explore the integration of NAA into multi-metabolite panels to enhance diagnostic robustness and counteract inter-patient heterogeneity. Additionally, a notable limitation of the current literature is that the overall scope of evidence remains concentrated on a relatively small number of solid tumors. The selection of malignancies included in this review is primarily dictated by the availability of existing empirical studies, whereas the role of the NAA/NAT8L axis in hematological malignancies remains virtually unexplored. Consequently, it is currently unclear whether the proposed metabolic and immunomodulatory concepts represent a generalized hallmark of cancer biology or are restricted to specific solid tumor lineages. Future studies extending into hematological malignancies and broader oncological contexts will be necessary to determine the generalizability of NAA axis dysregulation across cancer types.

Alongside these diagnostic considerations, therapeutic translation encounters its own set of challenges. First, tissue-specific delivery of NAT8L or ASPA modulators remains a major challenge. These enzymes also regulate normal adipose metabolism and whole-body energy balance. Systemic inhibition could therefore disrupt healthy tissues. Tumor-targeted delivery systems, such as antibody-drug conjugates or nanoparticle-based approaches, may help achieve selective drug accumulation. However, such strategies remain largely unexplored for this axis. Second, direct NAT8L inhibition could theoretically be bypassed by compensatory metabolic pathways. For example, cancer cells might utilize alternative acetyl-CoA sources or the parallel NAAG pool to sustain NAA production. These potential escape mechanisms warrant careful evaluation in future preclinical studies. Third, reliable pharmacodynamic biomarkers are needed to monitor target engagement and therapeutic response. Circulating NAA may potentially serve this purpose. However, systematic validation of NAA as a pharmacodynamic marker is currently lacking. Addressing these challenges will be essential to translate NAA-targeted strategies from preclinical discovery to clinical application.

In conclusion, while we have only begun to scratch the surface of the clinical and mechanistic complexities of the NAA axis, it is clear that this pathway represents a central node of metabolic plasticity. Fully unraveling the reasons behind its context-dependent roles will be a prerequisite for transitioning NAA-targeted therapies from the laboratory to the forefront of precision medicine. For now, however, mechanistic understanding should remain the immediate priority.

## Figures and Tables

**Table 1:** Human studies of NAA axis expression in cancer.

Cancer Type	NAA Concentration	NAA Axis Expression	Key Findings	Evidence Type	Reference
NSCLC	12.6 ± 4.6 μM in tumor, 120 ± 20 nM in plasma (GC-MS)	NAT8L ↑, NAA ↑	NAT8L overexpression in NSCLC drives NAA production and blood secretion.	Tissue and plasma metabolomics, Functional knockdown	[68]
IBC (SUM149 cells)	n.a. (LC-MS)	NAA ↑	NAA serves as a storage metabolite for acetate, promoting cell survival under resource-scarce conditions.	Cell-line metabolomics, Functional knockdown	[67]
HGSOC	63 ± 12 μM (GC-MS)	NAT8L mRNA ↑, NAA ↑	High NAA and NAT8L levels predict worse survival.	Tissue metabolomics, Transcriptomics data, Functional knockdown and, in vivo experimental model	[32]
Ovarian cancer	n.a. (LC-MS/MS)	NAA ↑	The NAA levels of ascitic fluid correlate with disease stage.	Ascitic fluid metabolomics, Transcriptomics data, Functional knockdown	[31]
CRPC	n.a. (metabolomics)	NAT8L mRNA ↑, NAA ↑	Increased NAA provides aspartate for nucleotide synthesis for tumor growth.	Tissue and serum metabolomics	[69]
HCC	n.a.	NAT8L ↓	Hindering NAT8L expression in HCC promotes cell proliferation.	Transcriptomics data, Functional knockdown, overexpression, and in vivo experimental model	[75]
Pan-cancer	n.a.	NAT8L mRNA ↑ in 6 cancers, ↓ in 12 cancers	NAT8L may be a prognostic and immunotherapeutic target across cancers.	Transcriptomics data	[74]

Abbreviations: NSCLC: Non-small cell lung cancer; GC-MS: Gas chromatography-mass spectrometry; NAT8L: N-acetyltransferase 8-like; NAA: N-Acetylaspartate; IBC: Inflammatory breast cancer; n.a.: no available; HGSOC: High-grade serous ovarian cancer; LC-MS/MS: Liquid chromatography-tandem mass spectrometry; CRPC: Castration-resistant prostate cancer; HCC: hepatocellular carcinoma. ↑, increase; ↓, decrease.

**Table 2:** Targeted NAA axis treatment in cancer.

Cells/Animals	Targeted Treatment	Key Findings	Reference
GSCs, OPCs (Oli-Neu)	Exogeneous NAA (100 μM) or NAAG (10 μM), medium change every 48 h, harvest 2,4,6 d	Promote GSCs growth and inhibit Oli-Neu differentiation	[70]
Human lung adenocarcinoma (A549), human hepatocellular carcinoma (JHH-4)	NAT8L knockdown	Inhibit proliferation of A549 and JHH-4 cells	[71]
NSCLC cell lines	NAT8L knockdown	Reduce intracellular and secreted level of NAA	[68]
IBC (SUM149)	RhoC knockdown	Inhibit NAA production and alter glutamine utilization	[67]
Ovarian cancer cell lines (HeyA8, A2780); ovarian cancer and melanoma mouse models	NAT8L knockdown	Reduce cell viability, proliferation and tumor growth	[32]
Neuroblastoma (SH-SY5Y)	Exogeneous NAA (4 mM, 72 h)	Induce neuronal differentiation and enhance sensitivity to chemotherapeutic agents	[76]
Ovarian cancer cell lines (OVCAR3, SKOV3); human monocytes	GS knockdown; exogeneous NAA (10 or 20 μM, 24 h)	Glutamine addiction drives macrophage M2 polarization via NAA, and promotes a protumoral microenvironment	[31]
Human malignant glioma (U87MG)	Exogeneous NAA (5 mM–160 mM)	Inhibit HDACs activity and regulate miRNAs to cause G1 arrest and apoptosis	[77]
HCC cell lines (HepG2, HuH7)	NAT8L knockdown	Increase cytosolic aspartate and promote cell proliferation by fueling purine synthesis	[75]
Human breast cancer cell lines (MDA-MB-231, MCF-7, BT-474, MDA-MB-468, SK-BR-3, T47D), human normal breast epithelial cell line (MCF-10A), human chronic myelogenous leukemia cell line (K562), mouse breast cancer cell lines (4T1, EO771), mouse glioma cell line (GL261), human NK-cell leukemia cell line (NK92)	NAT8L knockout	Prevent the acetylation of lamin A-K542 by PCAF, thereby restoring immune synapse integrity and killing efficiency	[79]
Macrophage cell lines (THP-1); NSCLC mouse models	SST (0.5 g/kg or 2 g/kg, i.g., starting on the 4th day after the lung cancer cell lines LLC injection, daily); exogeneous NAA (10 μM, 48 h)	SST modulates NAA-related immunometabolism to correct macrophage imbalances and enhance anti-tumor immunity	[80]

Abbreviations: GSCs: Glioma stem-like cells; OPCs: Oligodendrocyte progenitor cells; NAA: N-Acetylaspartate; NAAG: N-acetylaspartylglutamate; NAT8L: N-acetyltransferase 8-like; NSCLC: Non–small cell lung cancer; IBC: Inflammatory breast cancer; GS: Glutamine synthetase; HDACs: Histone deacetylases; HCC: Hepatocellular carcinoma; PCAF: p300/CBP-associated factor; SST: Shiyiwei Shenqi tablets; i.g.: Intragastric administration.

## Data Availability

No data were created or analyzed in this study.

## Abbreviations

The following abbreviations are used in this manuscript:

NAA	N-acetylaspartate
CNS	Central nervous system
NAT8L	N-acetyltransferase 8-like
ASPA	Aspartoacylase
RhoC	Ras homolog family member C
Acetyl-CoA	Acetyl coenzyme A
HDACs	Histone deacetylases
IBC	Inflammatory breast cancer
NAAG	N-acetylaspartylglutamate
CRPC	Castration-resistant prostate cancer
NSCLC	Non-small cell lung cancer
HGSOC	High-grade serous ovarian cancer
HCC	Hepatocellular carcinoma
SLC25A13	Solute carrier family 25 member 13
TME	Tumor microenvironment
NMDA	N-methyl-D-aspartate
TCA	Tricarboxylic acid
ATP	Adenosine triphosphate
GC-MS	Gas chromatography-mass spectrometry
LC-MS/MS	Liquid chromatography-tandem mass spectrometry
FOXM1	Forkhead box protein M1
mTORC2	Mammalian target of rapamycin complex 2
NK	Natural killer
GSCs	Glioma stem-like cells
OPCs	Oligodendrocyte progenitor cells
GS	Glutamine synthetase
PCAF	p300/CBP-associated factor
SST	Shiyiwei Shenqi tablets
